# Free-breathing non-contrast flow-independent cardiovascular magnetic resonance angiography using cardiac gated, magnetization-prepared 3D Dixon method: assessment of thoracic vasculature in congenital heart disease

**DOI:** 10.1186/s12968-021-00788-3

**Published:** 2021-07-19

**Authors:** Alexander Isaak, Julian A. Luetkens, Anton Faron, Christoph Endler, Narine Mesropyan, Christoph Katemann, Shuo Zhang, Patrick Kupczyk, Daniel Kuetting, Ulrike Attenberger, Darius Dabir

**Affiliations:** 1 Department of Diagnostic and Interventional Radiology, University Hospital Bonn, Venusberg-Campus 1, 53127 Bonn, Germany; 2 Quantitative Imaging Lab Bonn (QILaB), Bonn, Germany; 3 Philips Healthcare, Hamburg, Germany

**Keywords:** Congenital heart disease, Thoracic vasculature, Magnetic resonance angiography, Non-contrast enhanced magnetic resonance angiography, REACT, Flow-independent, Free-breathing

## Abstract

**Background:**

To evaluate a non-contrast respiratory- and electrocardiogram-gated 3D cardiovascular magnetic resonance angiography (CMRA) based on magnetization-prepared Dixon method (relaxation-enhanced angiography without contrast and triggering, REACT) for the assessment of the thoracic vasculature in congenital heart disease (CHD) patients.

**Methods:**

70 patients with CHD (mean 28 years, range: 10–65 years) were retrospectively identified in this single-center study. REACT-CMRA was applied with respiratory- and cardiac-gating. Image quality (IQ) of REACT-CMRA was compared to standard non-gated multi-phase first-pass-CMRA and respiratory- and electrocardiogram-gated steady-state-CMRA. IQ of different vessels of interest (ascending aorta, left pulmonary artery, left superior pulmonary vein, right coronary ostium, coronary sinus) was independently assessed by two readers on a five-point Likert scale. Measurements of vessel diameters were performed in predefined anatomic landmarks (ascending aorta, left pulmonary artery, left superior pulmonary vein). Both readers assessed artifacts and vascular abnormalities. Friedman test, chi-squared test, and Bland-Altman method were used for statistical analysis.

**Results:**

Overall IQ score of REACT-CMRA was higher compared to first-pass-CMRA (3.5 ± 0.4 vs. 2.7 ± 0.4, P < 0.001) and did not differ from steady-state-CMRA (3.5 ± 0.4 vs. 3.5 ± 0.6, P = 0.99). Non-diagnostic IQ of the defined vessels of interest was observed less frequently on REACT-CMRA (1.7 %) compared to steady-state- (4.3 %, P = 0.046) or first-pass-CMRA (20.9 %, P < 0.001). Close agreements in vessel diameter measurements were observed between REACT-CMRA and steady-state-CMRA (e.g. ascending aorta, bias: 0.38 ± 1.0 mm, 95 % limits of agreement (LOA): − 1.62–2.38 mm). REACT-CMRA showed high intra- (bias: 0.04 ± 1.0 mm, 95 % LOA: − 1.9–2.0 mm) and interobserver (bias: 0.20 ± 1.1 mm, 95 % LOA: − 2.0–2.4 mm) agreements regarding vessel diameter measurements. Fat-water separation artifacts were observed in 11/70 (16 %) patients on REACT-CMRA but did not limit diagnostic utility. Six vascular abnormalities were detected on REACT-CMRA that were not seen on standard contrast-enhanced CMRA.

**Conclusions:**

Non-contrast-enhanced cardiac-gated REACT-CMRA offers a high diagnostic quality for assessment of the thoracic vasculature in CHD patients.

## Background

Congenital heart disease (CHD) is the most common congenital disorder affecting about 0.8 % of life births [1]. Advances in diagnosis, treatment, and monitoring of CHD have led to a dramatic improvement of long-term survival [1, 2]. However, the clinical course of patients with CHD varies and late complications limit long-term clinical outcome [3, 4]. Therefore, life-long follow-up non-invasive imaging is indicated in CHD patients.

Due to its wide availability, noninvasiveness, and cost effectiveness, echocardiography is the first-line imaging modality in patients with CHD [5, 6]. However, its use is limited due to user dependency and a restricted acoustic window, especially for the assessment of vascular structures. As a radiation-free, reproducible, and standardized imaging modality cardiovascular magnetic resonance (CMR) became a mainstay of cardiovascular imaging [7]. Phase-contrast CMR imaging and contrast-enhanced CMR angiography (CMRA) have been implemented as essential components within the standard CMR protocol for the evaluation of the vascular system of patients with different types of CHD [6, 7].

Besides standard contrast-enhanced first-pass-CMRA with multiphase acquisition, which is performed without cardiac gating and during one breath hold, also contrast-enhanced respiratory- and electrocardiogram (ECG)-gated steady-state-CMRA—acquired during a steady-state of contrast enhancement—is used for thoracic vasculature imaging [8]. In two previous studies it was shown that steady-state-CMRA allows for significantly better image quality and additional diagnostic value in imaging the thoracic vasculature of CHD patients  compared to first-pass-CMRA [8, 9]. However, both procedures require the use of an extracellular contrast agent with low risk of complications such as extravasation, allergic reaction, or even extremely rare, nephrogenic systemic fibrosis [10]. Furthermore, the uncertainty regarding long-term effects of possible gadolinium retention in the brain after repetitive contrast-enhanced magnetic resonance imaging led to revised recommendations [11–13]. Also, mistiming of the contrast bolus can result in poor image quality. Consequently, non-contrast-enhanced CMRA techniques are desirable and have been developed for imaging of the thoracic vasculature, including steady-state free precession (SSFP) and balanced SSFP (bSSFP) techniques, spoiled gradient echo sequences, quiescent-interval single-shot, or fat-water separation Dixon-based methods [14–17]. However, the use of bSSFP techniques is limited due to pronounced banding artifacts. Furthermore, insufficient fat suppression and overall time-consuming acquisition when covering a large field-of-view are major drawbacks [18].

Recently, a free-breathing flow-independent 3D relaxation-enhanced angiography without contrast and triggering (REACT) has been introduced, which utilizes two magnetization-preparation pulses and a 3D dual-echo Dixon method [19]. This technique achieves robust suppression of static background tissue across a large field-of-view and enhances native blood vessels due to their difference in T1 and T2 relaxation times leading to good blood-to-tissue contrast [19].

The purpose of this study was to evaluate the feasibility and performance of a non-contrast-enhanced REACT-CMRA compared to standard contrast-enhanced first-pass- and steady-state-CMRA in imaging the thoracic vasculature of patients with different types of CHD.

## Materials and methods

### Study population

 The retrospective study was approved by the local institutional review board that waived informed consent. 72 CHD patients who underwent CMR including non-contrast-enhanced and contrast-enhanced CMRA in our department between September 2018 and November 2020 were identified. There were no exclusion criteria regarding the type of CHD, pathologies, or previous surgical procedures/interventions. Two patients had to be excluded subsequently due to distinct motion and respiratory artifacts in all three CMRA sequences.

### Imaging protocol

All examinations were performed on a clinical whole-body 1.5 T CMR system (Ingenia, Philips Healthcare, Best, Netherlands). For signal reception, a 32-channel torso coil with digital interface was used. The CMR protocol comprised ECG-gated bSSFP cine images in standard orientations (transversal, short-axis, four-chamber, three-chamber, two-chamber, left ventricular, and right ventricular outflow tract), and phase-contrast velocity-encoded flow imaging in vessels of interest. The multi-phase first-pass-CMRA was performed during breath-hold after intravenous administration of gadobutrol (Gadovist, Bayer Healthcare, Berlin, Germany) at a dose of 0.1 mmol/kg body weight and a flow rate of 1.5 ml/s, followed by a 20 ml saline flush using the same injection rate. The single-phase steady-state-CMRA was acquired during injection of 0.1 mmol/kg body weight of gadobutrol at a rate of 0.3 ml/s, also followed by a 20 ml saline flush, with ECG and respiratory navigator gating. Late gadolinium enhancement imaging in standard orientations was also performed. A detailed description of this protocol has been described previously [9].

Non-contrast-enhanced REACT-CMRA was recently introduced as a relaxation-based flow-independent sequence by Yoneyama et al. [19]. It is based on a T2-prep module (allowing for a higher arteriovenous contrast), a non-volume selective inversion recovery magnetization-preparation pulse (increasing the contrast between blood vessels and the surrounding area), and a 3D dual-echo Dixon-based acquisition (allowing for a robust residual fat suppression over a large field-of-view with good blood-to-tissue contrast). To minimize the influence of cardiac motion artifacts, ECG triggering was applied for end-diastolic acquisition. Images were acquired in end-expiration. A respiratory navigator with a fixed acceptance gating window (7 mm) was used to reduce respiratory motion artifacts.

**Fig. 1:**
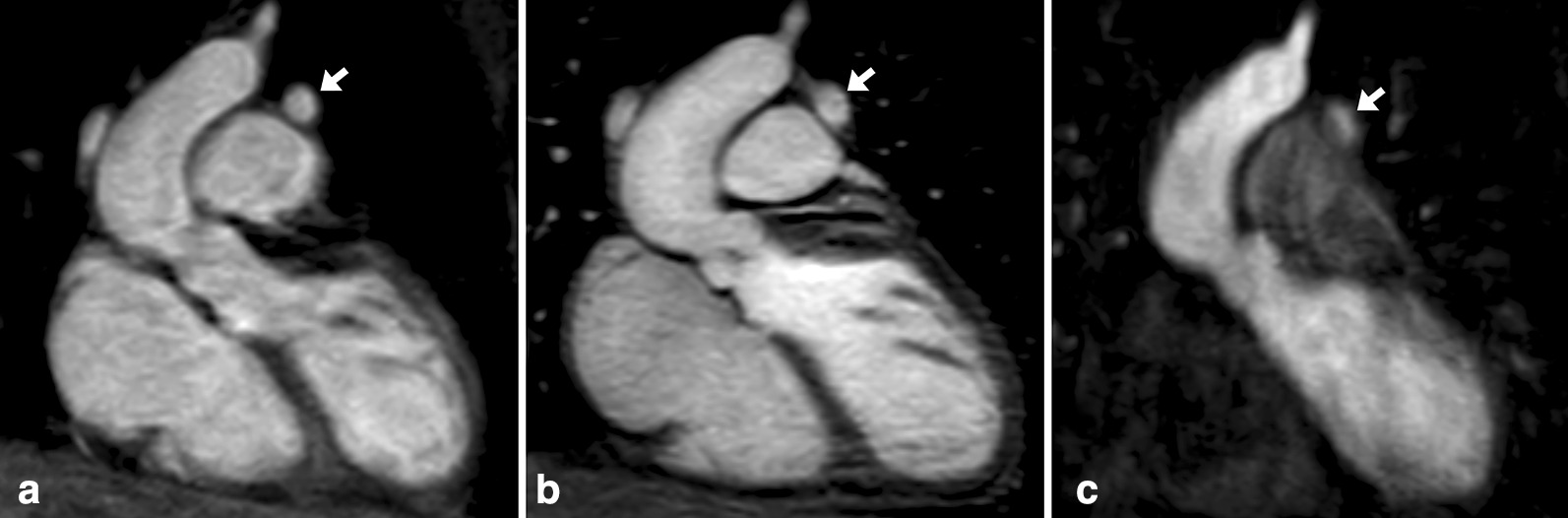
Images in coronal view and without multiplanar reformation in a 25-year-old female with surgical correction of tetralogy of Fallot. The presented images show non-contrast-enhanced relaxation-enhanced angiography without contrast and triggering (REACT) cardiovascular magnetic resonance angiography (CMRA) (**A**, water-only reconstruction), contrast-enhanced steady-state- (**B**), and first-pass-CMRA (**C**, arterial phase). Note the partially covered persistent left superior vena cava (arrow)

As a flow-independent technique, REACT-CMRA provides a simultaneous depiction of arterial and venous vessels. The reconstructed water-only images were used for analysis. REACT-CMRA was acquired before contrast injection. For imaging acceleration, parallel imaging with sensitivity encoding was used for all three CMRA techniques. All CMRA sequences were acquired in coronal orientation (Fig. 1). Detailed imaging parameters are given in Table 1.

**Table 1:** Imaging parameters of non-contrast-enhanced REACT cardiovascular magnetic resonance angiography (CMRA) and contrast-enhanced steady-state- and first-pass-CMRA

	REACT–CMRA	Steady–state–CMRA	First–pass–CMRA
Orientation	Coronal	Coronal	Coronal
Acquisition matrix (mm³)	252 × 285 × 188	288 × 194 × 120	352 × 240 × 70
Field of view (mm³)	400 × 457 × 150	360 × 360 × 150	400 × 370 × 126
Voxel size (mm³), acquired	1.6 × 1.6 × 1.6	1.25 × 1.85 × 2.5	1.14 × 1.54 × 3.6
Voxel size (mm³), reconstructed	0.8 × 0.8 × 0.8	0.8 × 0.8 × 1.25	0.76 × 0.76 × 1.8
T2 prep/inversion delay time (ms)	50/~70	–/270	–/–
Fat suppression	Dual–echo Dixon	–	Subtraction
TR/TE_1_/TE_2_ (ms)	5.8/1.72/3.8	3.2/1.01/-	3.5/1.13/-
Electrocardiogram gating	Yes	Yes	No
Turbo field echo factor	60	31	–
Respiratory navigator gating	Yes	Yes	No (breath–holding)
Flip angle (degree)	15	20	40
Parallel imaging (SENSE) factor	3.2	4	3.5
Nominal acquisition duration (min)	3:24	2:46	0:56

*REACT* relaxation-enhanced angiography without contrast and triggering, *TR* time of repetition, *TE* time of echo, *SENSE* sensitivity encoding

### Image analysis

Image quality (IQ) of non-contrast-enhanced REACT-CMRA, contrast-enhanced first-pass, and steady-state-CMRA was qualitatively and quantitatively assessed by two readers with 4 (AI, first reader) and with 11 years (DD, second reader) of CMR experience. Both readers independently evaluated the images using dedicated software (IMPAX EE, Agfa Healthcare, Bonn, Germany) in different sessions and on anonymized images. Both readers were blinded to the medical history in each case. The assessment of diagnostic value and the presence of susceptibility (e.g. related to stent implantation), flow (e.g. insufficiency jets), and fat-water artifacts (e.g.  misallocation artifact) was performed in consensus by both readers. For evaluation of REACT-CMRA the water-only images were used.

### Qualitative analysis

The ascending aorta (AAo), left pulmonary artery (LPA), left superior pulmonary vein (LSPV), coronary sinus (CS), and right coronary ostium (RCO) were defined as vessels of interest. Both readers separately rated IQ based on a five-point Likert scale: (1) non-diagnostic, (2) poor (severe artifacts, severe vessel blurring), (3) intermediate (some artifacts, some vessel blurring), (4) good (minimal artifacts, minimal vessel blurring, (5) excellent (no artifacts, good vessel border delineation).

### Vessel measurement

AAo, LPA, and LSPV were defined for measurement of the vessel diameter. Measurements were conducted separately by both readers on previously defined positions according to proposed recommendations [20]. For this purpose, multiplanar reconstructions were used to determine the maximum axial vessel diameter on appropriately angulated images. The first reader repeated all measurements in a second session after a minimum of 30 days.

### Statistical analysis

Prism (version 8.4.3; GraphPad Software, Inc., San Diego, California, USA) was used for statistical analysis. The Shapiro–Wilk test was applied for the assessment of normal distribution. Continuous characteristics are presented as mean ± standard deviation or as absolute frequency. Comparison of vessel measurements between the different CMRA sequences were compared by using one-way ANOVA followed by Tukey multiple comparison tests. Non-parametric Friedman test followed by Dunn test was used for multiple group comparison of image quality between the three applied CMRA techniques. Chi-squared test was used to compare the presence of non-diagnostic image quality. Bland–Altman analysis was used to evaluate differences in vessel measurements between non-contrast-enhanced and contrast-enhanced CMRA and also to determine intra- and interobserver reliability of vessel diameter measurements. The level of statistical significance was set to P < 0.05.

## Results

### General characteristics

A total of 70 subjects (43 males, 61 %; mean age: 28 ± 16 years, range: 10–65 years) were included in this study. The most common types of CHD were coarctation of the aortic isthmus (13/70, 18.6 %), tetralogy of Fallot (12/70, 17.1 %), congenital aortic valve dysplasia (7/70, 10.0 %), and dextro-transposition of the great arteries (5/70, 7.1 %). The complete list of the underlying types of CHD is presented in Table 2. The majority of patients received surgical treatment (52/70, 74.3 %).

**Table 2:** Indications for cardiovascular magnetic resonance

Leading pathology	Number of patients (n = 70)
Coarctation of the aortic isthmus	13 (18.6 %)
Tetralogy of Fallot	12 (17.1 %)
Congenital aortic valve dysplasia	7 (10.0 %)
Dextro–transposition of the great arteries (d–TGA)	5 (7.1 %)
Levo–transposition of the great arteries (l–TGA)	4 (5.7 %)
Ebstein’s anomaly	3 (4.3 %)
Hypoplastic left heart syndrome	3 (4.3 %)
Persistent truncus arteriosus	3 (4.3 %)
Pulmonary atresia	3 (4.3 %)
Congenital pulmonary stenosis	3 (4.3 %)
Ventricular septaldefect	3 (4.3 %)
Patent foramen ovale	2 (2.9 %)
Others*	9 (12.9 %)

Congenital heart diseases ranged from simple to complex/combined defects. For each patient, the leading pathology in the context of the underlying congenital heart disease is listed

* Other diagnoses include: Double outlet right ventricle, double-chambered right ventricle, congenital aortic aneurysm, sinus venosus defect with anomalous pulmonary vein drainage, tricuspid atresia, patent ductus arteriosus, congenital anomaly of superior vena cava, cor triatriatum sinistrum, scimitar syndrome (n = 1 per diagnosis)

### Sequence acquisition

The observed mean total scan time was 1:54 ± 0:25 min for multiphase first-pass-CMRA (including four phases), 6:06 ± 1:57 min for steady-state-CMRA, and 6:21 ± 1:59 min for REACT-CMRA (P < 0.001 for REACT- versus first-pass-CMRA and P = 0.52 for REACT-versus steadys-state-CMRA, respectively).

### Image quality

**Fig. 2:**
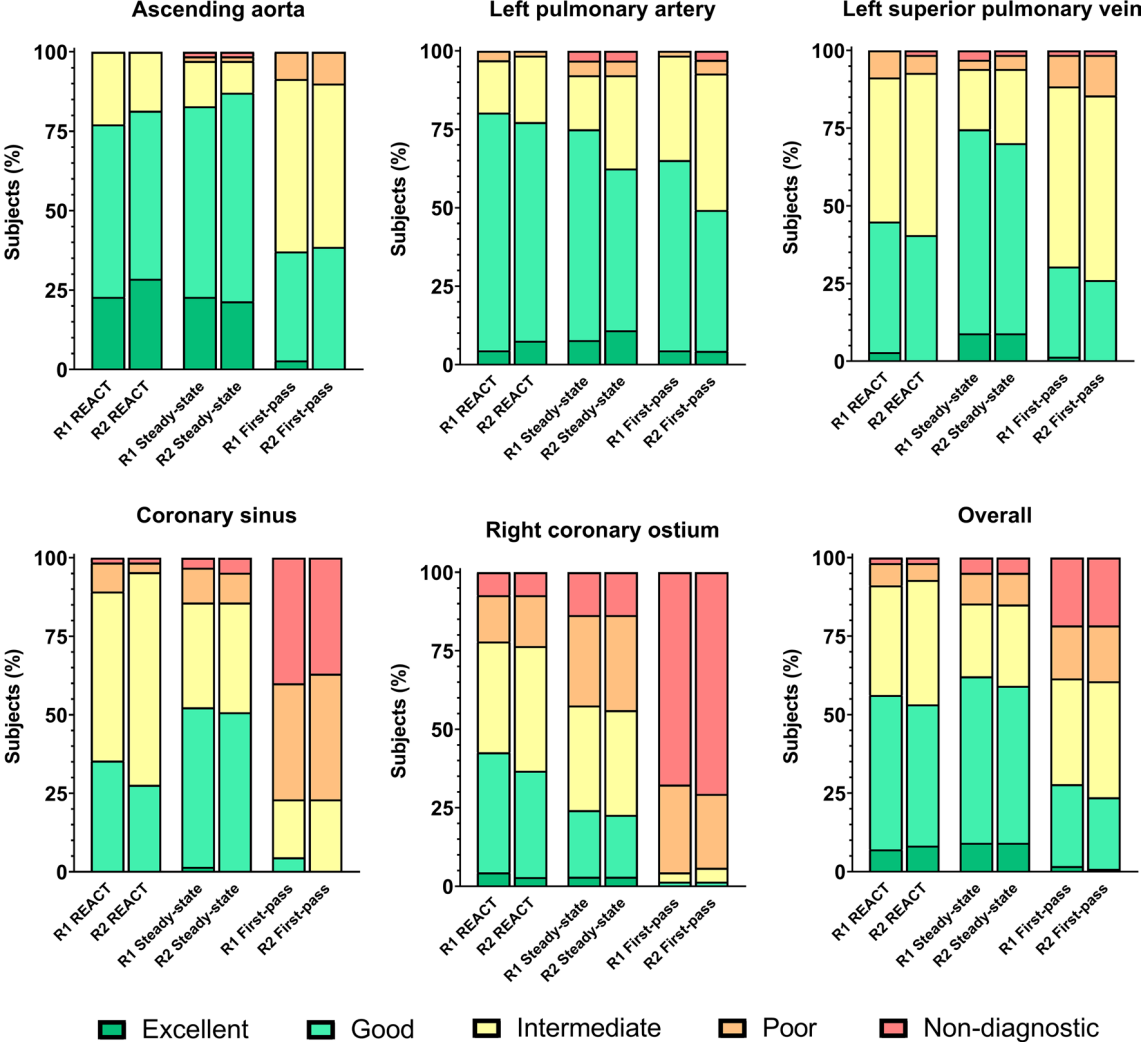
Bar-plots of image quality scores based on a five-point Likert scale of REACT-, steady-state-, and first-pass-CMRA. *R1* first reader, *R2* second reader

**Fig. 3:**
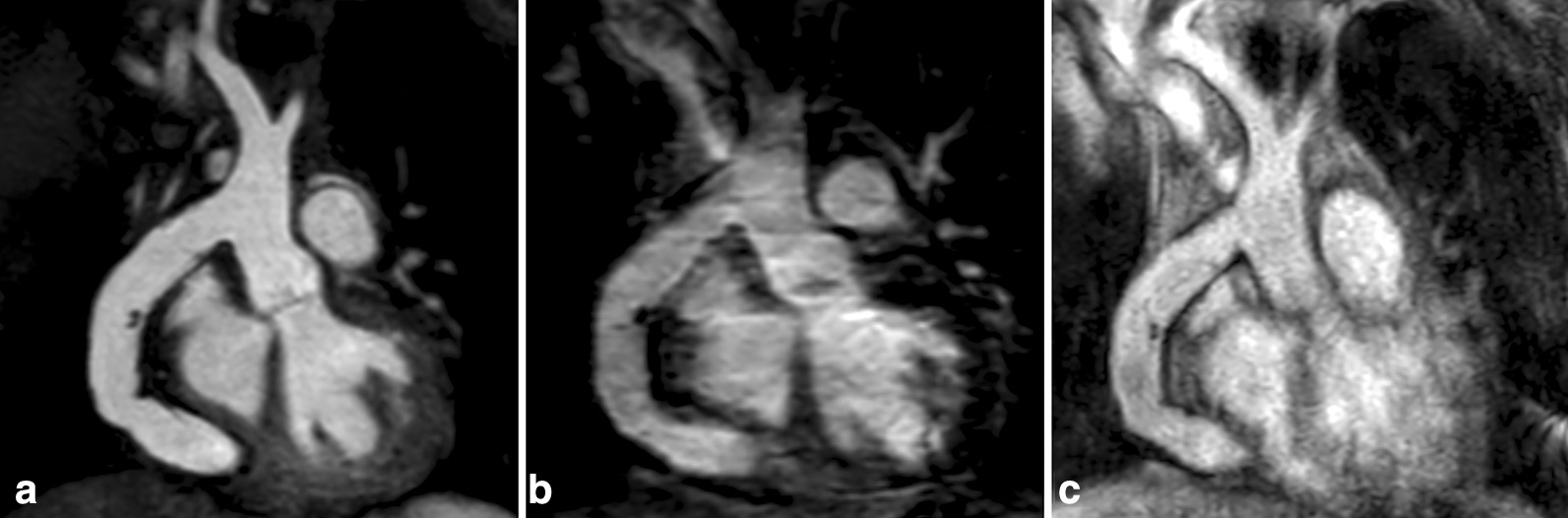
Images in coronal view and without multiplanar reformation in a 23-year-old female with an aorto-aortic conduit due to severe coarctation of the aortic isthmus. The presented example shows superior image quality of non-contrast-enhanced REACT-CMRA (**A**, water-only reconstruction) compared to contrast-enhanced steady-state- (**B**) and first-pass-CMRA (**C**) and provides a general impression of image quality over a large field-of-view. Note the clear delineation of fine suture material in the midline of the conduit on REACT-CMRA

IQ results of both readers are presented in Fig. 2. Comparisons of IQ scores are based on the average rating value of both readers and summarized in Table 3. A significant difference in overall IQ ratings (including all vessel measurements) was seen between REACT- and first-pass-CMRA (3.5 ± 0.4 vs. 2.7 ± 0.4, P < 0.001), but not between REACT- and steady-state-CMRA (3.5 ± 0.4 vs. 3.5 ± 0.6, P = 0.99). Evaluation of the AAo showed a high mean IQ of REACT- without a significant difference compared to steady-state-CMRA (4.1 ± 0.6 vs. 4.0 ± 0.7, P > 0.99), but with a significant difference compared to first-pass-CMRA (4.1 ± 0.6 vs. 3.3 ± 0.6, P < 0.001) (Fig. 3). Regarding the IQ of the LPA no significant difference was observed between all three CMRAs (Table 3). Steady-state-CMRA yielded the highest mean IQ score in evaluation of the LSPV but did not significantly differ from REACT-CMRA (3.7 ± 0.7 vs. 3.4 ± 0.6, P = 0.08). IQ evaluation of the CS showed a significant difference between REACT- and first-pass-CMRA (3.2 ± 0.6 vs. 1.9 ± 0.8, P < 0.001). The highest mean IQ score of the RCO was yielded by REACT-CMRA with significant difference compared to first-pass- (3.1 ± 0.9 vs. 1.4 ± 0.6, P < 0.001) and also steady-state-CMRA (3.1 ± 0.9 vs. 2.7 ± 1.0, P = 0.03).

**Table 3:** Comparison of mean image quality scores between non-contrast-enhanced REACT-CMRA and contrast-enhanced steady-state- and first-pass-CMRA

	REACT–CMRA	Steady–state–CMRA	First–pass–CMRA	*P* value
Ascending aorta	4.1 ± 0.6^†^	4.0 ± 0.7^†^	3.3 ± 0.6^‡‖^	< 0.001
Left pulmonary artery	3.8 ± 0.5	3.7 ± 0.8	3.6 ± 0.6	0.08
Left superior pulmonary vein	3.4 ± 0.6	3.7 ± 0.7^†^	3.1 ± 0.6^‡^	< 0.001
Coronary sinus	3.2 ± 0.6^†^	3.3 ± 0.8^†^	1.9 ± 0.8^‡‖^	< 0.001
Right coronary ostium	3.1 ± 0.9^†‡^	2.7 ± 1.0^†‖^	1.4 ± 0.6^‡‖^	< 0.001
Overall	3.5 ± 0.4^†^	3.5 ± 0.6^†^	2.7 ± 0.4^‡‖^	< 0.001

Variables are given as mean ± standard deviation. P values were obtained using Friedmann test followed by Dunn multiple comparison test

^‡^ P < 0.05 versus steady-state-CMRA

^†^ P < 0.05 versus first-pass-CMRA

^‖^ P < 0.05 versus REACT-CMRA

A non-diagnostic IQ was observed in 6 out of 350 (1.7 %; CS: 1, RCO: 5) evaluated vessels on REACT-CMRA, in 15/350 (4.3 %; AAo: 1, LSPV: 1, LPA: 1, CS: 2, RCO: 9) vessels on steady-state-CMRA (P = 0.046 versus REACT-CMRA), and in 73/350 (20.9 %; LSPV: 1, CS: 26, RCO: 46) vessels on first-pass-CMRA (P < 0.001 versus REACT-CMRA).

### Vessel measurements

**Fig. 4:**
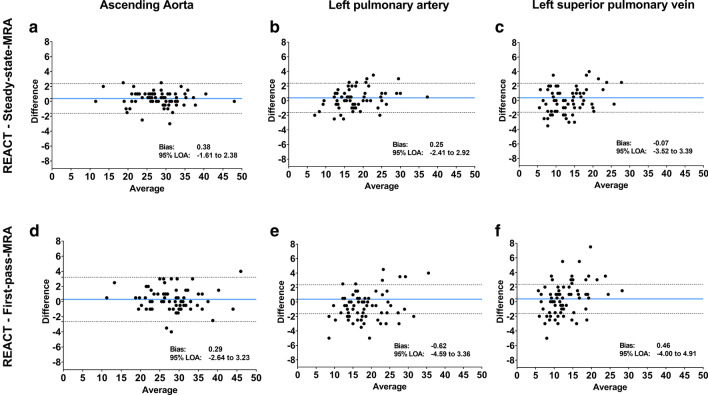
Bland–Altman comparison of vessel diameter measurements (in mm) on representative thoracic vessels (ascending aorta, left pulmonary artery, and left superior pulmonary vein) by the first reader (A.I.). Figures show comparisons between non-contrast-enhanced REACT-CMRA, contrast-enhanced steady-state- (**A**–**C**), and first-pass-CMRA (**D**–**F**), respectively. The middle line depicts the mean bias of the diameter measurements and the dotted lines represent the 95 % limits of agreement (LOA)

AAo, LPA, and LSPV showed similar mean vessel diameters without significant differences between REACT- and steady-state- or first-pass-CMRA (Table 4). Bland–Altman comparisons of vessel diameter measurements showed closer 95 % limits of agreement (LOA) between REACT- and steady-state-CMRA compared to REACT- and first-pass-CMRA (Fig. 4).

**Table 4:** Averaged vessel diameter measurements of both reviewers on non-contrast-enhanced REACT-CMRA, contrast-enhanced steady-state-, and first-pass-CMRA

	REACT–CMRA	Steady–state–CMRA	First–pass–CMRA	*P* value
Ascending aorta (mm)	28.2 ± 6.1	27.7 ± 6.0	27.9 ± 6.3	0.96
Left pulmonary artery (mm)	18.3 ± 6.0	17.7 ± 5.5	18.8 ± 5.5	0.84
Left superior pulmonary vein (mm)	13.2 ± 5.2	13.1 ± 4.8	12.7 ± 4.4	0.80

Vessel diameters are given as mean ± standard deviation. P values were obtained using one-way ANOVA followed by Tukey multiple comparison tests

### Intra- and interobserver agreement

**Fig. 5:**
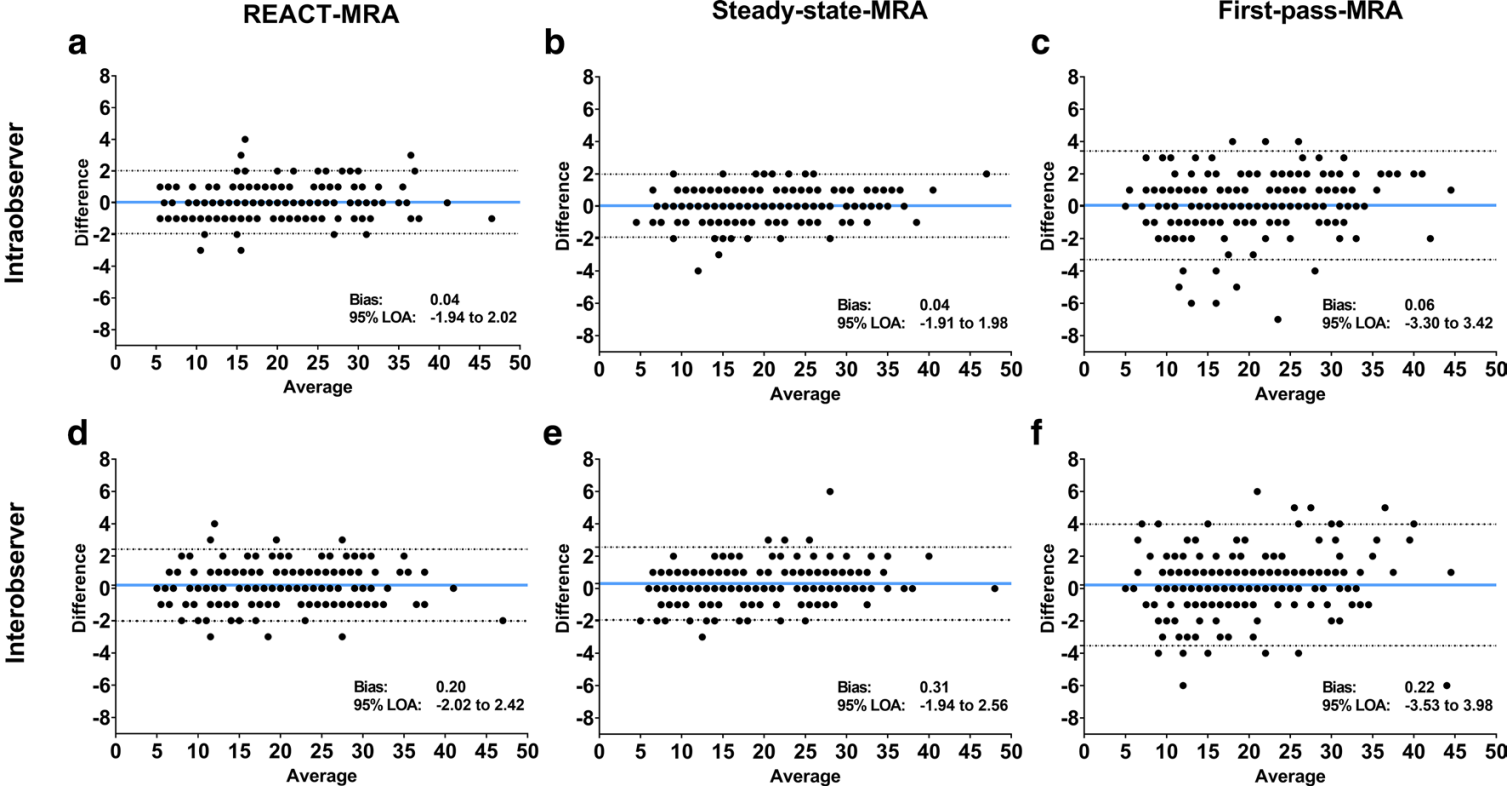
Bland–Altman comparison of all vessel diameter measurements (in mm) show intra- (**A**–**C**) and interobserver (**D**–**F**) agreement on non-contrast-enhanced REACT-CMRA and contrast-enhanced steady-state- and first-pass-CMRA. The blue line depicts the mean bias of the diameter measurements and the dotted lines represent the 95 % limits of agreement (LOA)

For intra- and interobserver agreement, Bland–Altman comparisons revealed closer 95 % LOA for REACT- and steady-state-CMRA compared to first-pass-CMRA (Fig. 5). Intraobserver reliability including all vessel diameter measurements was 0.04 ± 1.0 mm (95 % LOA: − 1.9–2.0 mm) on REACT-CMRA, 0.06 ± 1.7 mm (95 % LOA: 3.3–3.4 mm) on first-pass-CMRA, and 0.04 ± 1.0 mm (95 % LOA: − 1.9–2.0) on steady-state-CMRA, respectively. Interobserver reliability was 0.20 ± 1.1 mm (95 % LOA: − 2.0–2.4 mm) on REACT-CMRA, 0.22 ± 1.9 mm (95 % LOA: − 3.5–3.9 mm) on first-pass-CMRA, and 0.31 ± 1.1 mm (95 % LOA: − 1.9–2.6 mm) on steady-state-CMRA, respectively.

### Diagnostic value

**Fig. 6:**
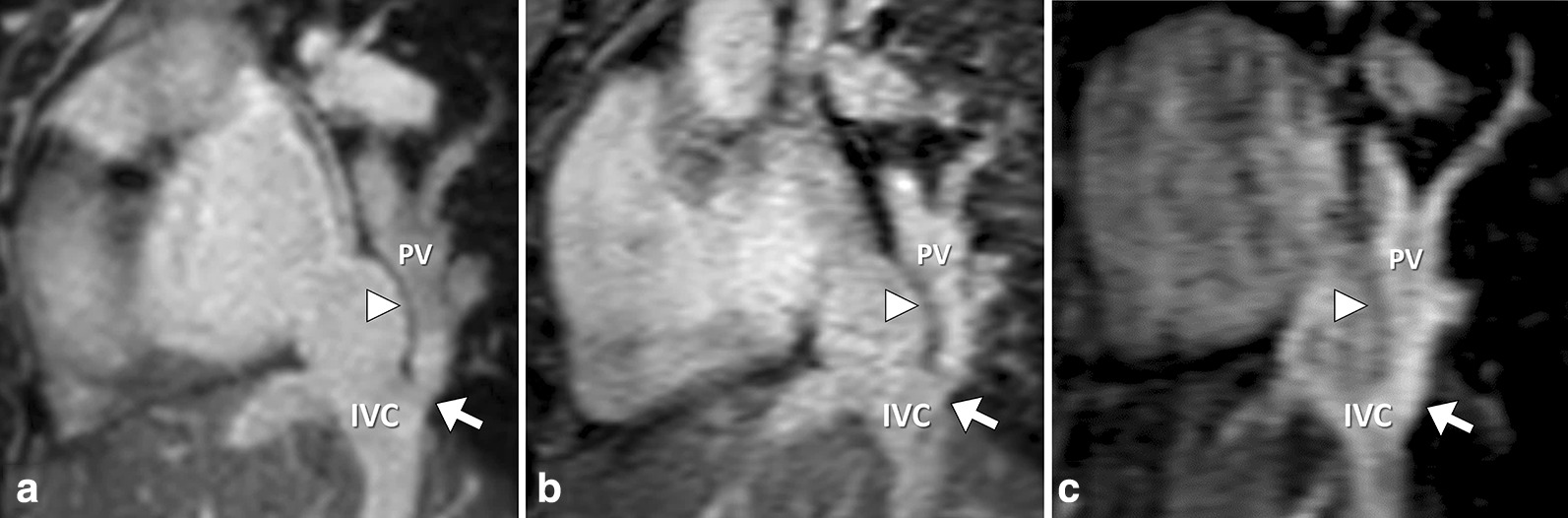
52-year-old female patient with scimitar syndrome. Representative multiplanar reformatted images. REACT-CMRA (**A**, water-only reconstruction) clearly shows an anomalous venous return from both right pulmonary veins (PV) to the inferior vena cava (IVC) rather than directly to the left atrium (junction marked by the arrow). Steady-state-CMRA (**B**) shows intermediate image quality with blurred contours and some vessel artifacts. First-pass-CMRA (**C**) offers a good contrast but shows distinct artifacts and a bad demarcation between the PV and IVC (arrowhead) in comparison to the sharp demarcation on REACT-CMRA (arrowhead)

**Fig. 7:**
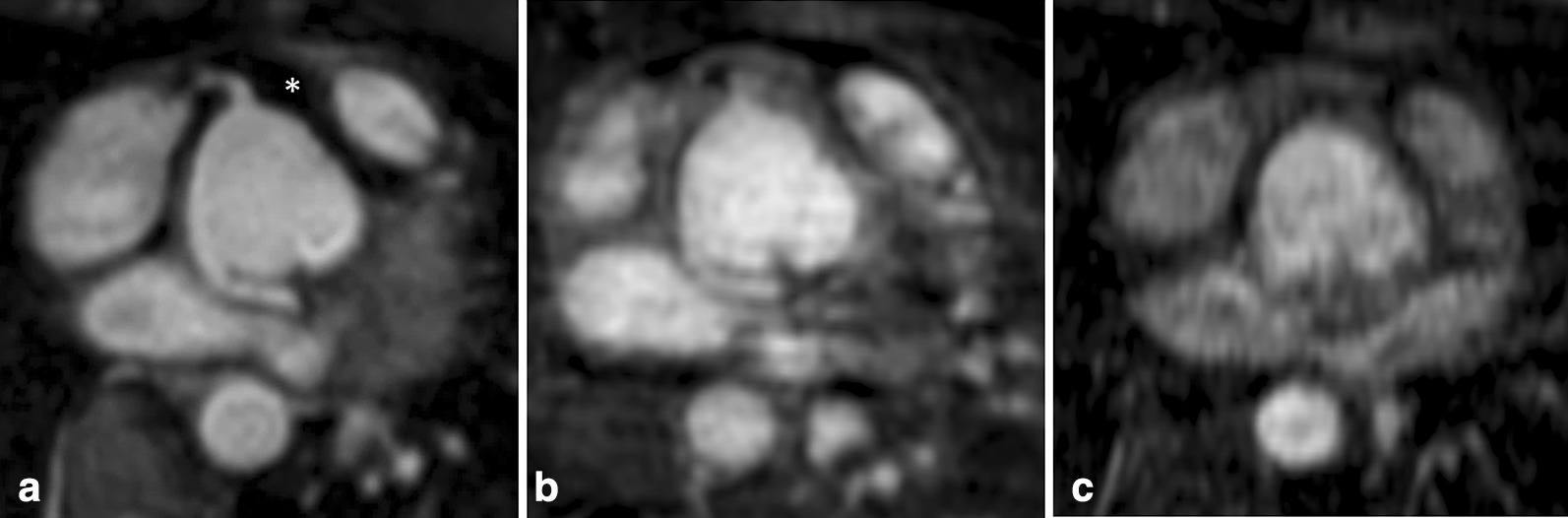
Multiplanar reformatted images of the coronary ostia in a 30-year-old female patient with surgical correction of persistent truncus arteriosus (type I). Non-contrast-enhanced REACT-CMRA (**A**, water-only reconstruction) shows good image quality with homogeneous epicardial fat suppression (asterisk), allowing for precise delineation of the proximal right coronary artery and aberrant origin of the left coronary artery from the non-coronary sinus. On contrast-enhanced steady-state-CMRA (**B**) the right coronary ostium is only rudimentarily recognizable and the left coronary artery shows blurred contours with surrounding artifacts. First-pass-CMRA (**C**) is non-diagnostic regarding the coronary arteries

**Fig. 8:**
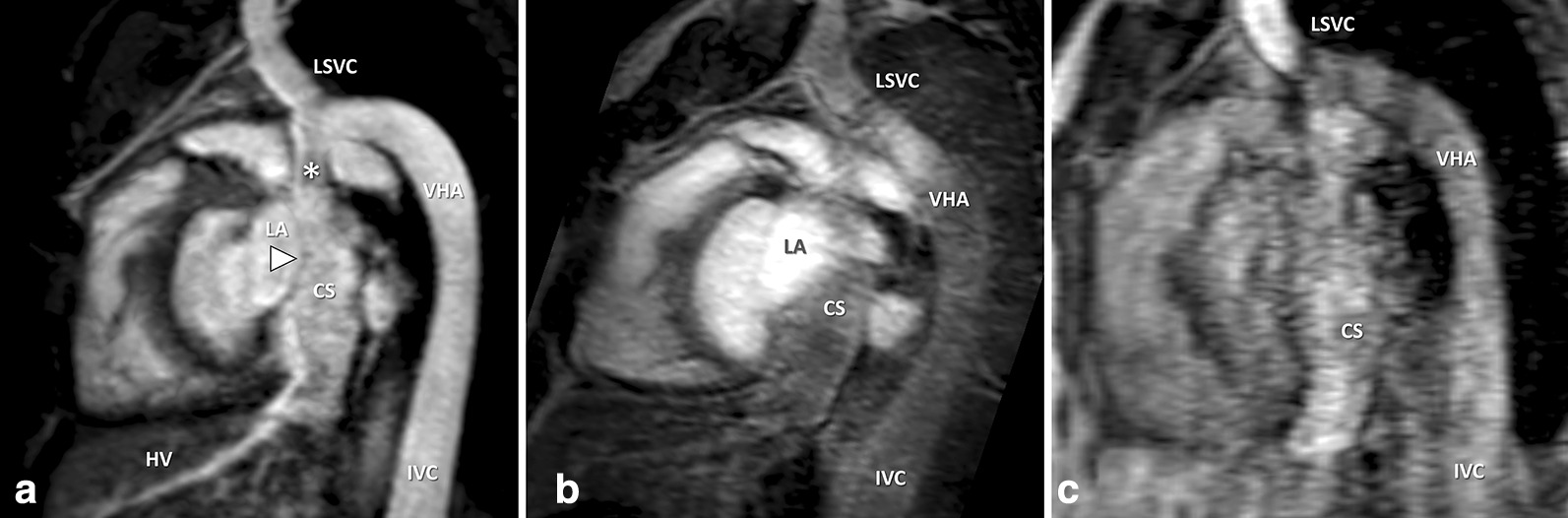
Multiplanar reformatted images in a 29-year-old male patient with venous anomaly: inferior vena cava (IVC) with hemiazygos continuation to the persistent left superior vena cava (LSVC), draining into the dilated coronary sinus (CS). Right SVC was also present without communicating vein. The hepatic veins (HV) were directly connected to the CS. REACT-CMRA (**A**, water-only reconstruction) offered good image quality, with clear visualization of the confluence (star) between persistent LSVC, vena hemiazygos (VHA), and the CS. Steady-state-CMRA (**B**) was non-diagnostic due to insufficient contrast within the anomalous veins. First-pass-CMRA (**C**) showed pronounced vessel artifacts with blurred image contours. Note the fine delineation between the left atrium (LA) and the CS (arrowhead), without visible fenestration which is only clearly evaluable on REACT-CMRA images

Non-diagnostic IQ regarding the RCO was seen in 5/70 patients on REACT-CMRA (7 %; steady-state-CMRA: 9/70, 13 %; first-pass-CMRA: 46/70, 66 %). REACT-CMRA improved the diagnostic value by more precise differentiation of even small vessels from directly adjacent structures due to high resolution and less blurring (Fig. 6). REACT-CMRA enabled the detection of an anomalous coronary ostium in 3 cases, which would have been missed by steady-state- and first-pass-CMRA only (Fig. 7). Additionally, a fusiform aneurysmatic dilatation of the proximal right coronary artery was visualized by REACT-CMRA, but not by the applied contrast-enhanced CMRAs. In one case diagnosis of cor triatriatum sinistrum could be established by REACT-CMRA (clear delineation of the abnormal septation within the left atrium and concomitant flow jet), but not by means of steady-state- and first-pass-CMRA alone. Opposed to the contrast-enhanced CMRAs, REACT-CMRA allowed for the correct diagnosis of a partial anomalous pulmonary venous connection and a complex venous anomaly in one patient (Fig. 8). In total, six additional vascular abnormalities were revealed by REACT-CMRA compared to contrast-enhanced CMRAs.

### Artifacts

**Fig. 9:**
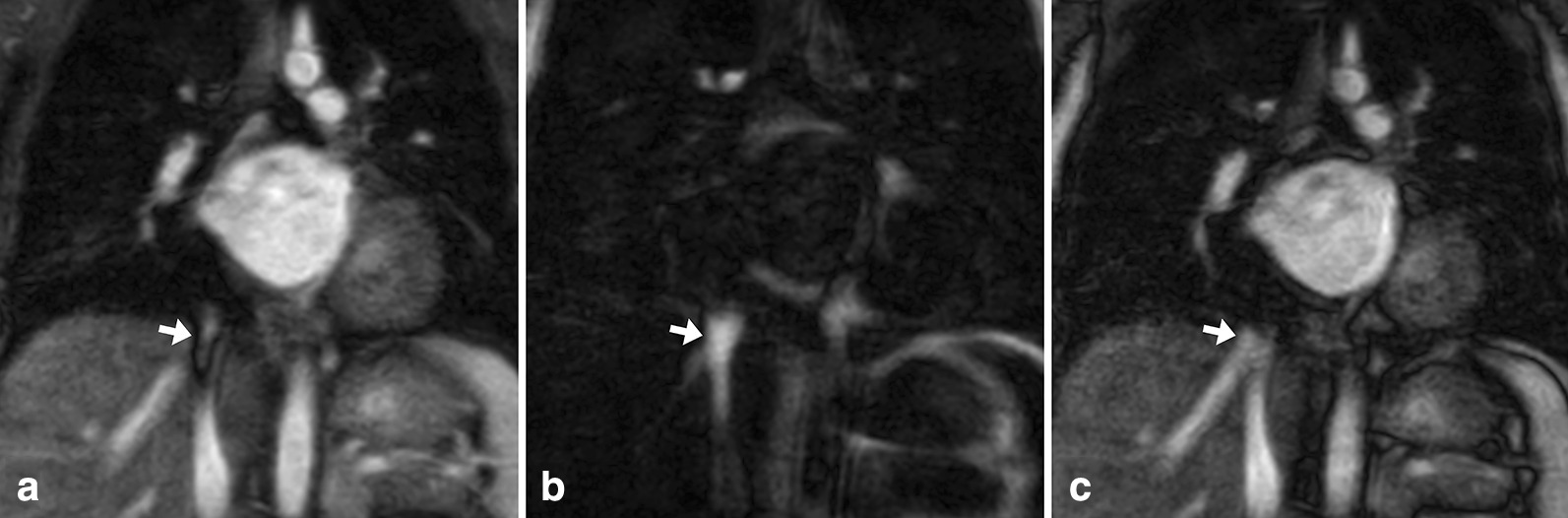
REACT-CMRA images in coronal view show a flow-related fat-water separation artifact in typical location, which is commonly observed in dual modified Dixon-based sequences. In the water-only image (**A**) a hypointense rimed artifact (arrow) is seen within the distal IVC. Fat-only images (**B**) show high signal in the same location, indicating a signal swapping (the signal of moving water appears in the fat images most likely due to phase shifts between the two echoes). In this case, the in- and out-of-phase images should also be reconstructed, because they usually show regular artifact-free signal, as shown in the presented out-of-phase image (**C**). Knowledge of the presented artifact is important, as it may mimic vena cava inferior or hepatic vein thrombosis

**Fig. 10:**
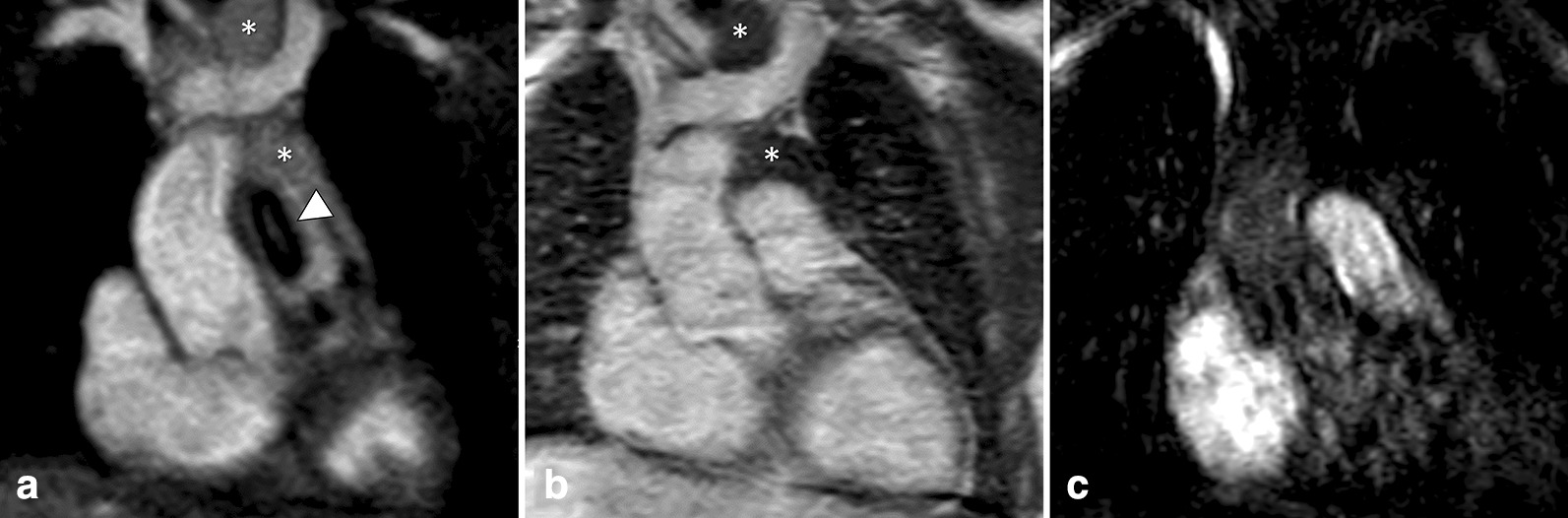
10-year-old male patient with surgical correction of tetralogy of Fallot (non-reformatted coronal view). A flow-related artifact (most likely due to incorrect fat-water separation) is seen on non-contrast-enhanced REACT-CMRA (**A**, water-only reconstruction; fat-only and in- and opposed phase reconstructions are not available) within the main pulmonary artery (arrow), but neither on contrast-enhanced steady-state-CMRA (**B**) or first-pass-CMRA (**C**). Phase-contrast flow measurement revealed moderate pulmonary valve stenosis (pulmonary valve maximum blood velocity: 3.1 m/s). Background suppression of thymus tissue was more efficient on steady-state- than REACT-CMRA (asterisks)

Susceptibility artifacts were the most frequent encountered artifacts and affected each of the three CMRAs (14/70 patients, 20 % for each CMRA). They were mainly related to surgical or interventional procedures. Furthermore, flow artifacts were present in 10/70 patients (14 %) on REACT- and steady-state-CMRA, respectively, but not on first-pass-CMRA. Fat-water separation artifacts were observed on REACT-CMRA in 11/70 cases (16 %), which are specific to chemical shift encoding sequences and Dixon methods [21, 22]. However, some of these artifacts are known to appear on water- and fat-only images and can be circumvented by the additional reconstruction of in- and opposed-phase images. In the majority of cases this type of artifact was observed on REACT-CMRA in regions with high or turbulent flow, as a manifestation of an inappropriate allocation of signal in water- and fat-images [23]. A common location for the signal misallocation artifact on REACT-CMRA was the transition of the vena cava inferior and the right atrium (Fig. 9). In five patients (7 %) distinct flow artifacts in the evaluated vessels were observed on REACT-CMRA. Although the image quality was reduced in these cases, the occurrence of this artifact did not lead to a diagnostic misinterpretation compared to first-pass- and steady-state-CMRA. Rather, the encountered artifact did also provide diagnostic utility regarding hemodynamic pathologies, e.g. moderate or severe stenosis or insufficiency (Fig. 10). Furthermore, bright signal of stagnant fluid (e.g. pericardial or periaortic fluid) due to long T1 relaxation times was observed on REACT-CMRA, which led to impaired vascular delineation in four cases (6 %).

## Discussion

In this study, we compared non-contrast-enhanced respiratory navigated and ECG-gated REACT-CMRA to conventional contrast-enhanced non-gated multi-phase first-pass-CMRA and contrast-enhanced respiratory navigated, ECG-gated steady-state-CMRA for assessment of the thoracic vasculature in a wide spectrum of CHD. We were able to show that the implementation of REACT-CMRA is feasible and yields precise vessel delineation, even in cases of complex cardiovascular anatomy as frequently observed in patients with CHD. The overall IQ of REACT-CMRA did not significantly differ from high-resolution contrast-enhanced steady-state-CMRA and was significantly higher compared to contrast-enhanced first-pass-CMRA. Vessel measurements of REACT-CMRA showed good intra- and interobserver agreement without difference compared to the standard contrast-enhanced sequences. Although artifacts were observed on REACT-CMRA, the overall artifact burden was low and did not limit diagnosis of the underlying disease. In some cases, REACT-CMRA showed additional diagnostic value compared to the established contrast-enhanced methods due to better background suppression and improved vessel delineation.

CMRA techniques are an important component in almost every CMR protocol for initial diagnostic work-up or follow-up of children and adults with CHD. However, the application of a gadolinium-based contrast-agent is needed for the acquisition of standard CMRA sequences. Gadolinium-based contrast agents have a favorable tolerance and severe complications are rare since the introduction of new-generation agents. However, based on recent studies on gadolinium deposition in the brain, the restrained use of contrast agents is recommended due to uncertain long-term effects, especially in young patients [24, 25]. Moreover, peripheral intravenous cannulation is often traumatic in children; therefore, contrast-free examinations can increase patient compliance. In fact, patients with CHD are predominantly young and require regular follow-up CMR examinations throughout their lives; therefore contrast-free techniques are desirable in this cohort. Over the last years, different non-contrast-enhanced CMRA techniques have been established, which may contribute to lower examination costs and facilitate clinical workflow. Several ECG- and respiratory-gated sequences, mainly based on SSFP, bSSFP, spoiled gradient echo, quiescent-interval single-shot or modified Dixon techniques, were introduced to assess the thoracic vasculature, particularly the coronary arteries (commonly known as “whole heart” sequences) [26–28]. Although flow-independent bSSFP-based techniques offer high signal- and contrast-to-noise ratios, they are often affected by an inadequate fat suppression as well as flow- and banding-artifacts due to off-resonance effects [29]. In a recent study, a modified Dixon-based CMRA showed fewer artifacts compared to bSSFP sequence [27]. However, the major limitation of these CMRA sequences is the time-consuming acquisition when using a large field of view, e.g. a scan time of up to 10 min for a pulmonary vein imaging 3D bSSFP CMRA or about 7.4 min for a 3D mDixon based thoracic CMRA [27, 30].

Recently, REACT-CMRA was introduced by Yoneyama et al. [19]. This new pulse sequence enables free-breathing acquisition of both arterial and venous vessels with high spatial resolution and effective fat suppression across a large field-of-view. It is based on two magnetization-preparation pulses (T2-prep pulse and non-selective inversion recovery pulse) for suppression of background tissue and enhancement of native blood due to differences in T1 and T2 relaxation times, which consequently yields a robust blood-to-tissue contrast. Moreover, a dual-echo Dixon method is used for effective fat suppression over a large field-of-view. REACT-CMRA can be used in a variety of applications, e.g. for imaging of the pelvic veins, extracranial arteries, or the thoracic vasculature [22, 31, 32]. To compensate for cardiac and respiratory motion artifacts, respiratory navigator- and ECG-gating was applied in this study. As REACT-CMRA is a flow-independent technique, both arterial and venous vessels can be assessed at the same time. Though simultaneous acquisition of the thoracic vasculature is time-efficient, it can be disadvantageous compared to multiphase-CMRA, when small arteries and veins are localized close together and a clear delineation is not possible. However, a reliable vessel delineation is normally ensured by high spatial resolution, as known from contrast-enhanced steady-state-CMRA. Recently published studies of Pennig et al. could show improved imaging quality of respiratory navigator- and ECG-gated Dixon-based CMRA for imaging the pulmonary vessels compared a to a 4D contrast-enhanced CMRA in a cohort of 25 patients [32]. Furthermore, improved image quality in imaging the aorta was seen using a modified REACT-CMRA in comparison to a single-phase contrast-enhanced CMRA [33]. Our results could also show a significant better image quality of REACT-CMRA compared to the untriggered breath-hold first-pass-CMRA, which mainly is related to the use of ECG- and respiratory gating and leading to a lower occurrence of artifacts and a sharper vessel delineation. Furthermore, mDixon method enabled robust background suppression, especially of the epi- and pericardial fat. Beyond that, we could show that overall image quality of REACT-CMRA did not significantly differ from ECG- and navigator-gated steady-state-CMRA, which is commonly used for high-resolution imaging of the thoracic vasculature in patients with CHD—but is dependent on contrast agent administration.

A non-diagnostic image quality level was seen in only 1.7 % of all evaluated vessels on REACT-CMRA versus 4.3 % on steady-state-CMRA and even 20.9 % on first-pass-CMRA (most hereby affected vessel regions were CS and RCO). Moreover, a direct diagnostic benefit was yielded by the additional use of REACT-CMRA. The use of REACT-CMRA improved the detection of the proximal coronary arteries and showed additional diagnostic value in individual cases of complex cardiovascular conditions due to good blood-to-tissue contrast, high spatial resolution, and effective fat suppression of the epicardial fat. The mean scan time of REACT-CMRA was comparable to steady-state-CMRA and prolonged compared to first-pass-CMRA. However, the additional use of compressed sensing can be potentially used for accelerating image acquisition [32]. The overall burden of artifacts on REACT-CMRA imaging was low, but areas with high flow velocities or severe insufficiency were affected in a few cases. Despite the reduced local image quality in these cases, artifacts also contained diagnostic information by indicating vascular or valvular pathologies (e.g. aortic stenosis, pulmonary stenosis, or aortic regurgitation). In our experience, the occurrence of water-fat separation artifacts of REACT-CMRA, which are commonly known in dual-Dixon-based sequences, could be effectively circumvented by reconstructing in- and opposed-phase besides water-only images [21, 23]. Furthermore, the high signal of stagnant fluid on REACT-CMRA may cause confusion if directly adjacent to the thoracic vessels and therefore should be kept in mind for careful interpretation.

### Clinical applications

Based on our study results and clinical experience, we can recommend the use of contrast-free REACT-CMRA in patients with CHD. Besides its use as a valuable supplementary sequence to visualize complex anatomic structures, REACT-CMRA can be a great trade-off to assess the thoracic vasculature in children who require repetitive follow-up CMR examinations without the need of contrast agents. Other suggested areas of applications may be patients with pregnancy, severe renal dysfunction, connective tissue disease, or general follow-ups, e.g. for simple assessment of aortic or pulmonary artery diameters.

It should be noted that unlike the standard time resolved first-pass-CMRA, REACT-CMRA - like steady-state-CMRA - provides only static information about the thoracic vessels. However, since vascular stenoses in patients with CHD almost exclusively affect vessels close to the heart and corresponding information is obtained quantitatively by phase contrast flow measurements as standard, this limitation is negligible for the vast majority of cases. Fat-separation and flow-related artifacts may occur and should be known to avoid misinterpretation.

### Limitations

Our study has limitations. First, the readers were not blinded to the CMRA sequences, which might have influenced observer bias. Second, due to a lack of standards, the image quality of non-contrast-enhanced techniques including REACT-CMRA may vary across institutions depending on the imaging parameter or even the CMR system. Third, no direct comparison to other non-contrast CMRA techniques like SSFP and bSSFP was made, as the current study focused on comparison to the clinical standard. A direct comparison to other non-contrast CMRA techniques is nevertheless useful and should be considered for future studies. Fourth, the comparison between non-ECG gated and ECG gated techniques is generally limited. Furthermore, digital subtraction angiography as the reference standard was not available. Fifth, there is a wide age range in the study cohort. Since non-contrast enhanced techniques are especially desirable in children a specific pediatric cohort of patients with CHD would be useful. Further studies are necessary to address these questions.

## Conclusions

REACT-CMRA enables contrast-free and reliable imaging of the entire thoracic vasculature in patients with CHD while providing higher image quality compared to the commonly used first-pass-CMRA and similar image quality compared to high-resolution contrast-enhanced steady-state-CMRA. REACT-CMRA is not only a useful alternative to standard contrast-enhanced CMRA but may also represent a decisive step toward contrast-free thoracic vasculature imaging in CHD patients.

## Data Availability

The datasets generated and/or analyzed during the current study are not publicly available due data protection but are available from the corresponding author on reasonable request.

## Abbreviations

AAo: Ascending aorta
bSSFP: Balanced steady-state free precession
CHD: Congenital heart disease
CMR: Cardiovascular magnetic resonance
CMRA: Cardiovascular magnetic resonance angiography
CS: Coronary sinus
ECG: Electrocardiogram
HV: Hepatic vein
IQ: Image quality
IVC: Inferior vena cava
LA: Left atrium
LOA: Limits of agreement
LPA: Left pulmonary artery
LSPV: Left superior pulmonary vein
PV: Pulmonary vein
RCO: Right coronary ostium
REACT: Relaxation-enhanced angiography without contrast and triggering.
SSFP: Steady state free precession
SVC: Superior vena cava
VHA: Vena hemiazygos
